# Non-ergodic dissociative valence double ionization of SF_6_

**DOI:** 10.1038/s41598-025-06972-0

**Published:** 2025-07-01

**Authors:** Emelie Olsson, Veronica Daver Ideböhn, Måns Wallner, Richard J. Squibb, John H. D. Eland, Ewa Erdmann, Raimund Feifel

**Affiliations:** 1 Department of Physics, University of Gothenburg, Origovägen 6B, 412 58 Gothenburg, Sweden; 2 Department of Chemistry, Physical and Theoretical Chemistry Laboratory, Oxford University, South Parks Road, Oxford, OX1 3QZ UK; 3 Faculty of Applied Physics and Mathematics, Gdańsk University of Technology, Narutowicza 11/12, 80-233 Gdańsk, Poland

**Keywords:** Atomic and molecular interactions with photons, Electronic structure of atoms and molecules

## Abstract

The dissociative double ionization of sulphur hexafluoride, SF_6_, in the ionization energy range from threshold up to 48.4 eV has been examined in detail using a multiple coincidence electron-ion technique. The results are interpreted by comparison with molecular dynamics simulations, high level molecular structure calculations and with a statistical model of the ion breakdown. Comparison between the experimental breakdown pattern and the pattern derived on the basis of statistical theory indicates that the energy redistribution required for fully statistical behaviour is incomplete on the timescale of the dissociation reactions of ${\text{SF}}_6^{2 + }$, suggesting that the molecular size at which ergodic behaviour becomes dominant is larger for doubly and multiply charged ions than for neutral and singly ionized molecules.

## Introduction

Sulphur hexafluoride, SF_6_, is a highly symmetric molecule of both fundamental interest and great practical importance. Because of its uses as a gaseous dielectric and insulator as well as in plasma etching processes, and its role as an atmospheric pollutant, the spectroscopy and dissociation dynamics of SF_6_ in different states of charge and excitation have been studied quite extensively, using photon excitation (e.g. Ref.^1^ and refs. therein) and electron beam excitation (e.g. Ref.^2^ and refs. therein). Previous work reported in the literature up to 2004 was reviewed in the paper on the complete valence double ionization spectrum of Feifel et al.^1^ using electron spectroscopy only. More recent work has concentrated mainly on the dynamics of its singly-positive ion dissociations^3,4^, most notably by double photodetachment of $\hbox {SF}_6^-$ species^4^. Kinetic energy release distributions, partly from dissociations of its doubly-positive ions were also measured following ionization by electron impact^3^, and one study focused specifically on the dissociative states of $\hbox {SF}_4^{2+}$ and the momentum distributions of the fragments^5^.

Apart from that, the fate of doubly positively charged SF_6_ has received little recent attention and is the subject of the present work. From ion-ion coincidence studies on processes induced by photon impact^6–8^ and by fast ion impact^9^ we already have an idea of the overall mechanistic pathways in nascent $\hbox {SF}_6^{2+}$ fragmentations. From electron-electron coincidence spectra induced by photon impact^1,10,11^ and from double charge transfer experiments^12^ augmented by theoretical calculations we have a basic understanding of the electronic structure of $\hbox {SF}_6^{2+}$ ions. To elaborate on that, in the present work, double photoionization of SF_6_ is investigated by means of multi-electron-multi-ion coincidence spectrosopy, yielding fragment-selected electron pair double ionization spectra. These are combined into a complete energy-dependent breakdown diagram and a set of experimental appearance potentials (AEs) and kinetic energy releases (KERs) for comparison with theoretical predictions obtained using density functional theory (DFT). To gain further insight into the dissociation dynamics, potential energy surface (PES) exploration and molecular dynamics (MD) simulations are complemented by calculations based on statistical theory, specifically Microcanonical Metropolis Monte Carlo (M_3_C) simulations^13,14^.

The M_3_C approach, by its independence of initial activation mechanisms, can make extensive characterization of possible fragmentation pathways. The fundamental assumption of fully statistical theories of unimolecular decay is that all energy in a molecule or ion is freely redistributed among energetically accessible parts of the phase space before reaction takes place. This means, for example, that all electronic excitation energy must be degradable to vibrational energy on the lowest potential energy surface. The range of molecular species, particularly positive ions, for which this assumption is strictly valid was examined several years ago in connection with the statistical theory of mass spectrometry. Clear cases where it is invalid (“isolated states”) were discovered in several molecular singly-charged ions by coincidence methods similar to the present work, or by observation of optical emission in competition with fragmentation, and are discussed in the book by Illenberger and Momigny^15^. In general, diatomic and triatomic species are clearly excluded from statistical treatment as their density of states is too low, but a molecule with seven atoms like SF_6_ might reasonably be expected to behave largely statistically. However, a disproportionate number of known isolated states were found in fluorinated systems, including the 8-atom $\hbox {C}_2\hbox {F}_6^+$ species^16^. Furthermore, while the timescale of internal vibrational redistribution is of the order of $10^{-12}$ seconds, charge separation times of doubly-charged ions may be similar because of the bond breakage on loss of two electrons and the impetus given by Coulomb repulsion. Thus the dissociation of $\hbox {SF}_6^{2+}$ is expected to be an intermediate case, where the question of statistical versus non-statistical behaviour must arise prominently. We believe that this is the first attempt to apply fully statistical theory to breakdown of a doubly-charged ion of modest size. By comparing the experimentally observed fragmentation pattern of the molecule with statistical predictions, we hope to elucidate the dissociation characteristics of $\hbox {SF}_6^{2+}$, and possibly provide a benchmark for the applicability of statistical methods to dissociations of doubly charged ions.

## Results and discussion

### Experimental

**Fig. 1:**
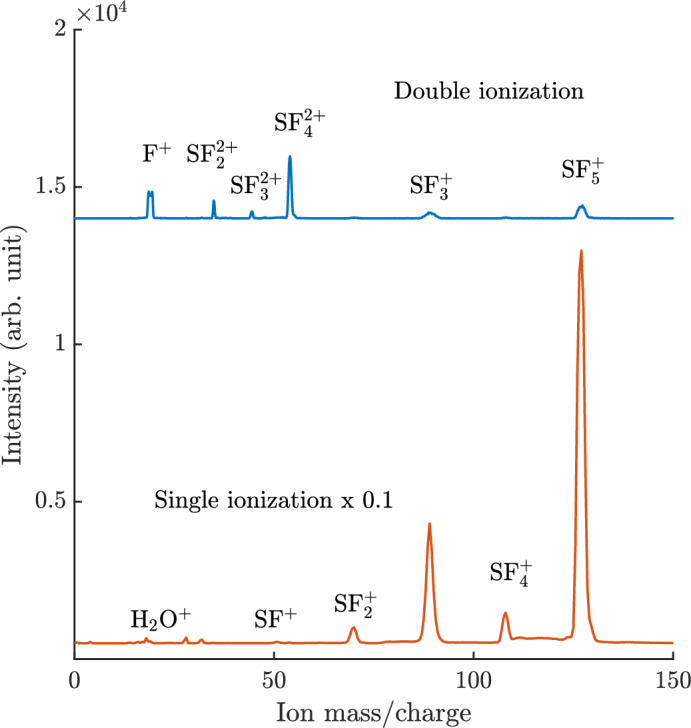
Pure single and double ionization mass spectra of SF_6_ at 48.4 eV photon energy. The spectra have been extracted by selection on electron numbers and energies corresponding to single and double ionization energy transfers, respectively. The intensities of the single ionization spectrum has been scaled by a factor of 1/10.

Single and double ionization mass spectra of SF_6_ obtained at 48.4 eV photon energy are shown in Fig. 1. On the basis of the well-known conventional valence photoelectron spectrum^17^, the single ionization mass spectrum was isolated by selecting single electrons in the binding energy range of 15–28 eV, which implies a limitation to the outer valence cationic states of SF_6_ up to the F $^2\hbox {A}_{1\textrm{g}}$ state. This excludes a portion of the single ionization spectrum below the double ionization onset which is essentially unstructured, and because of its low intensity it is unlikely to change the single ionization mass spectra significantly.

The double ionization mass spectrum is isolated by selection of electron pairs giving an energy transfer (h$\nu -$($\hbox {E}_1$+$\hbox {E}_2$)) above 37 eV, starting from below the double ionization onset of 37.5 eV^1^ up to 48.4 eV. For the extraction of the double ionization mass spectrum, random accidental coincidences have been subtracted, which was not necessary for the single ionization spectrum because of the much higher intensity of real events. The single ionization spectrum has been scaled in intensity by a factor of 1/10, but the intensities of the two spectra should not be compared directly since the electron collection-detection efficiency of the second electron required in double ionization has not been taken into account explicitly. Self-consistency within the double ionization mass spectrum is reflected in the reasonable agreement in total intensities between the F^+^ peak, which is partially split by the kinetic energy release (KER), and the summed intensities of the $\hbox {SF}_\textrm{n}^+$, (n = 2–5) peaks. This indicates that the detection efficiency for ions of different mass does not vary significantly over the range involved.

As can be seen in Fig. 1, the strongest fragmentation channel for nascent (but unobserved) $\hbox {SF}_6^{2+}$ is the formation of $\hbox {SF}_4^{2+}$, either together with neutral F_2_ or two fluorine atoms. Among four-fold coincidence events, from the charge separating processes, the strongest channel is $\hbox {SF}_3^{+}$ + F^+^, followed by $\hbox {SF}_5^{+}$ + F^+^. Compared to the $\hbox {SF}_4^{2+}$ channel, $\hbox {SF}_3^{+}$ + F^+^ is about half as abundant. Comparison of the upper and lower panels of Fig. 1 shows that the F^+^ ion is formed almost exclusively in double ionization, as already noticed in electron-impact studies^3,18^. Apart from that, we note that on the time-scale of our ion time-of-flight spectrometer, we could not identify any trace of a stable doubly-charged or singly charged parent ion nor of the possible ion $\hbox {SF}_5^{2+}$.

**Fig. 2:**
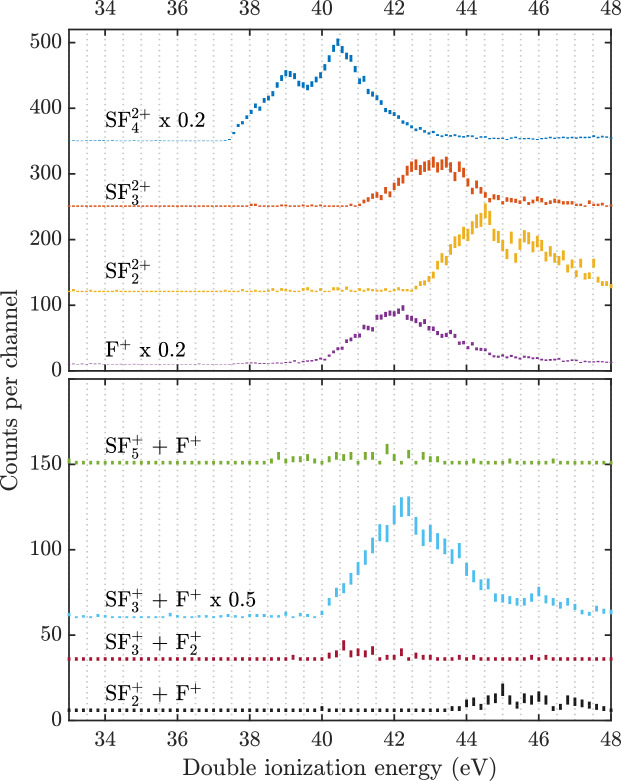
Upper panel: electron pair spectra based on threefold coincidence events for the main charge retaining channels and for production of F^+^ as proxy for all charge separation channels. Lower panel: electron pair spectra based on fourfold coincidence events (two electrons, two ions) showing the main charge separation channels, all displayed on the double ionization energy scale. The intensities for some of the spectra have been scaled by the factors given in the figure, and are displaced vertically for clarity. The error bars represent the statistical uncertainty of the coincidence counts.

To investigate how the energy transferred to $\hbox {SF}_6^{2+}$ leads to the different decay channels, threefold electron-electron-ion coincidence events are analysed for the charge-retaining reactions, and fourfold coincidence events with two electrons and two ions for the charge separating channels. Figure 2 reflects the abundances of the major charge retaining pathways in the top four spectra, and the charge separating decays in the lower four spectra, as a function of the double ionization energy. As F^+^ is created in all the strong charge separations, its intensity serves as a proxy to represent their summed intensity on the same scale as the charge retaining ones. The charge separations are dominated by the $\hbox {SF}_3^{+}$ + F^+^ channel. Also, as shown, the $\hbox {F}_2^+$ fragment is formed primarily with $\hbox {SF}_3^{+}$, either directly from $\hbox {SF}_6^{2+}$ or from an undetected intermediate $\hbox {SF}_5^{2+}$.

**Fig. 3:**
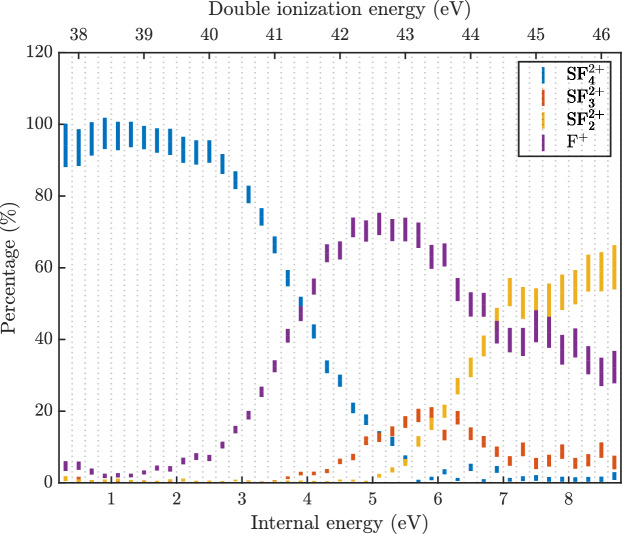
Breakdown diagram for the decay channels of $\hbox {SF}_6^{2+}$, where F^+^ is taken as a proxy for all charge separating channels. The lower horizontal axis represents internal energy in nascent $\hbox {SF}_6^{2+}$ as explained in the text, and the upper horizontal axis gives the double ionization energy. The error bars are based on propagation of the statistical uncertainty in the individual spectra.

Figure 3 shows a breakdown diagram, where the observed doubly charged ions are represented directly and F^+^ represents the summed intensity of all the charge separation channels (as in Fig. 2). The lower horizontal axis gives the internal energy of $\hbox {SF}_6^{2+}$ above its assumed lowest state at 37.5 eV and the upper axis is the double ionization energy. At low internal energies only double charge retention is observed, and at the highest energies it starts to dominate the intensity again (cf. $\hbox {SF}_2^{2+}$ yield), which is unexpected. Its apparent re-emergence could be partly due to loss of charge separating fragments at high double ionization energies, where the kinetic energy release is expected to be higher and the ions are more likely to be lost.

The relative abundances, appearance energies (AEs), shoulder and peak energies from the different dissociation channels in Fig. 2 are summarized in Table 1. The relative abundances were determined using the threefold or fourfold intensities, taking the ion collection-detection efficiency into consideration, and setting the most intense channel $\hbox {SF}_4^{2+}$ to 100. The rightmost column lists the KERs for the charge separating decays, extracted from the twofold ion-ion coincidences. The $\hbox {SF}_4^{+}$ + F^+^ channel is sufficiently intense for us to extract the KER from the peak width including all electron energies at this photon energy, but the peak width as a function of ionization energy, which would require fourfold coincidences, cannot be extracted because there are too few counts available.

$\hbox {SF}_4^{2+}$ + 2F/F_2_ appears at the very onset of double ionization, followed by $\hbox {SF}_5^{+}$ + F^+^ at 38.5 eV. The other channels all start at higher ionization energies and persist after the $\hbox {SF}_4^{2+}$ channel seems to close at about 43 eV. The first clear onsets are considerably lower than those determined by Frasinski et al.^19^, and are more in line with those of Joachims et al.^20^, both using photoionization. The double ionization threshold of Griffiths and Harris^12^ at 38.9 eV based on charge-exchange experiments is also considerably higher than our onset value, but roughly matches the centre of the first double ionization peak. Previous theoretically calculated double ionization thresholds^1,9,12^ cover a range of energies both above and below the onset we observe here.

In this context, it is relevant that both the conventional photoelectron spectrum^17^ and the threshold electron spectrum^10^ show that an “$\hbox {F2s}^{-1}$” inner valence ionization band spans the double ionization onset and extends up to at least 45 eV. It is therefore expected that a significant fraction of double ionization upon single photon impact at short wavelength occurs indirectly, through intermediate formation of singly-charged inner valence states. This means that the nuclear positions and molecular symmetry at the moment of double ionization are not necessarily restricted to the Frank-Condon region defined by the ground-state geometry of the neutral molecule. It is thus unsurprising that different forms of ionization and also photoionization at different photon energies will give different apparent double ionization thresholds.

**Table 1:** Fragmentation channels, relative abundances and appearance energies (AEs) of doubly-ionized SF_6_ using 48.4 eV photons, together with shoulder and peak double ionization energies, as well as kinetic energy release (KER) in the charge separating channels. The uncertainty of the double ionization electron spectra related energies is ± 0.5 eV, and for the KER it is about ± 0.3 eV.

Ion(s)	Relative abundance	AE (eV)	Shoulder (eV)	Peak (eV)	KER (eV)
$\hbox {SF}_4^{2+}$	100	37.4	38.1	39.0, 40.4	
$\hbox {SF}_3^{2+}$	8	41.0	41.5	42.5–43.4	
$\hbox {SF}_2^{2+}$	15	42.6	43.3	44.5	
$\hbox {F}^{+}$	62	38.5	41.1	41.5–42.3	
$\hbox {SF}_5^{+}$ + F^+^	2.4	38.5		41.8	4.7
$\hbox {SF}_3^{+}$ + F^+^	50	40.0	41.6	42.0–42.4, 46.0	4.2
$\hbox {SF}_3^{+}$ + $\hbox {F}_2^+$	1.7	40.2		40.6	4.5
$\hbox {SF}_2^{+}$ + F^+^	3	44.0		45.0	2.6
$\hbox {SF}_4^{+}$ + F^+^					4.1

Compared to the complete, electron-only double ionization electron spectrum of Feifel et al.^1^, the $\hbox {SF}_4^{2+}$ yield spectrum in Fig. 2 contains more marked resolved features, particularly in the range where charge separations compete for the overall intensity. This suggests that the decay pathway to this fragment is initiated by ionization out of a particular subset of the molecular orbitals of the parent compound. The lowest energy states populated at the photon energy of 48.4 eV were interpreted with the help of Green’s function calculations^1^ as states from initial removal of two 2p electrons from F atoms located as far apart as possible (distal F atoms) followed by states from removal of one electron from a F 2p orbital and one from the S atom. Peaks in the theoretical spectrum of summed pole strengths come at roughly 2 eV intervals, similar to the spacing of features in the here presented experimental $\hbox {SF}_4^{2+}$ electron pair spectrum.

Ion-ion coincidence maps can provide important additional information on the pathways for some decays, as the slopes of ion-ion correlation islands can often indicate the likely mechanism. Conservation of linear momentum requires that charge separation to two bodies creates an island of slope -1, while non-unit slopes may result from reactions where three or more fragments separate. If an initial singly-charged fragment dissociates further after separating from its partner ion, the ideal slope of the resulting island can be calculated from the masses of the fragments. In the present case, the $\hbox {SF}_4^{+}$ + F^+^ island has a slope of −0.9, which is in good agreement with the slope of −0.85 expected from the sequential pathway $\hbox {SF}_6^{2+} \rightarrow$ $\hbox {SF}_5^{+}$ + F^+^ and $\hbox {SF}_5^{+} \rightarrow$ $\hbox {SF}_4^{+}$ + F, and also in agreement with results from electron-impact ionization studies^5^. In contrast to this pathway with the neutral fragmentation in the second step, the $\hbox {SF}_3^{+}$ + $\hbox {F}_2^+$ island has a slope of −1, which is expected from the charge separating decay of $\hbox {SF}_5^{2+}$, whereas if the reaction was sequential via $\hbox {SF}_4^{+}$ the ideal slope would be −0.65. These two islands can be seen in Fig. S1 of the Supplementary Materials.

For the charge separating decays, the KERs of the dissociations were estimated based on the widths of the ion-ion coincidence peaks and are listed in Table 1. The uncertainty of the KER values is about ± 0.3 eV. We note that the present values are in agreement with those of Eland and Treves-Brown^8^, and also Bapat et al.^5^ for the $\hbox {SF}_3^{+}$ + F^+^ channel. For the $\hbox {SF}_3^{+}$ + $\hbox {F}_2^+$ channel, the third fragment is one neutral atomic F, and if, as discussed in the previous section the immediate parent is $\hbox {SF}_5^{2+}$, then neutral evaporation must have occurred before the charge separating decay. The KER in the neutral F evaporation $\hbox {SF}_6^{2+} \rightarrow$ $\hbox {SF}_5^{2+}$ + F, from the broadening across the $\hbox {SF}_3^{+}$ + $\hbox {F}_2^+$ ion-ion coincidence island, is about 0.6 eV. Peak broadenings such as this caused by priorly neutral evaporations have been subtracted from the peak widths when extracting the KER values presented in Table 1 when possible.

Kinetic energy values were also estimated for dissociations following single ionization, and compared with the more precise velocity map imaging based results of Bull et al.^3^. These authors measured the kinetic energy distributions for individual fragments following electron-impact ionization using electrons of 100 eV energy, yielding mostly singly charged fragments from single ionization, but with contribution from double ionization. The initial states of nascent singly or doubly ionized SF_6_ populated by electron-impact and photoionization are expected to be much the same, as is also discussed in Ref.^3^.

We note that our method of estimating the KER from the FWHM of the ion time of flight peaks provides a mean value for the KER, and underestimates it slightly. For comparison of KERs to Bull et al.^3^ we have calculated the kinetic energy of the individual fragments, and not the total KER in each dissociation. For individual singly charged fragments $\hbox {SF}_\textrm{n}^{+}$, our kinetic energy values are 0.4, 0.2, 0.1, 0.1 eV for n = 2–5, respectively, in good agreement with those of Bull et al.^3^ considering the uncertainty of our estimates. Bull et al.^3^ also provide kinetic energy distributions from double ionization for the doubly charged fragments $\hbox {SF}_4^{2+}$, $\hbox {SF}_3^{2+}$ and $\hbox {SF}_2^{2+}$, where our individual mean kinetic energies of $\sim$0.1 eV for each of them are in agreement. For these double charge retaining fragments, the total broadening from several neutral F/F_2_ evaporations is similar for the three doubly charged ions, which is not surprising since the evaporation can occur in different directions.

Further inspection of the $\hbox {SF}_4^{2+}$ ion time of flight peak width as a function of double ionization energy suggests the presence of two distinct KERs. It is evident that the peak width increases step-wise when comparing the KERs for the double ionization energy ranges 37.5–39.5 eV and 40–41.1 eV, corresponding to the first peak including the shoulder and solely the second peak, respectively. Accounting for the thermal width of the parent ion, and assuming the neutral fragment to be F_2_, the mean total KERs for the dissociation in the two double ionization ranges are 0.3 and 0.6 eV, respectively. The absolute values of these KERs are quite uncertain, but indicate the order of magnitude.

The immediate origin of the $\hbox {SF}_3^{+}$ + F^+^ channel is known from earlier ion-ion coincidence measurements to be two-body charge separation of $\hbox {SF}_4^{2+}$. It is shown not only by unit slope of the coincidence island, seen in Fig. S1 of the Supplementary Materials, but also by an unusually intense metastable “tail” in the ion-ion coincidence map^8^. Relatively slow dissociations (ns-$\mu$s lifetimes) give rise to a diagonal tail in the coincidence map, while faster dissociations (shorter than ns lifetimes) build the main $\hbox {SF}_3^{+}$ + F^+^ coincidence peak. This metastable decay of $\hbox {SF}_4^{2+}$ appears as a weak but clear plateau at masses below the $\hbox {SF}_3^{+}$ peak in the coincident mass spectrum. Selection of this plateau allows the double ionization electron spectrum of the metastable process itself to be extracted. It has very much the same shape as the $\hbox {SF}_3^{+}$ + F^+^ spectrum itself in Fig. 2, meaning that a range of the same internal energies of $\hbox {SF}_6^{2+}$ lead to both slow and fast dissociations. The apparent independence of the metastable’s appearance on the initial internal energy of $\hbox {SF}_6^{2+}$ is remarkable, as slow metastable decays normally arise only from the lowest internal excitation energies of a precursor. If it is assumed that only the least internally excited $\hbox {SF}_4^{2+}$ species decay slowly, then up to 4 eV of the original internal excitation energy would have been taken away from $\hbox {SF}_6^{2+}$ by departing neutral fragments or by photon emission. To be consistent with the measured KERs (< 1 eV), two neutral fluorine atoms might leave with high velocity in different directions. Because the first excited state of the F atom is at a too high energy, electronic excitation of the products is not possible^21^. The $\hbox {SF}_4^{2+}$ and $\hbox {SF}_3^{+}$ + F^+^ ion peaks both show the same KER broadening from neutral F evaporation, indicating once again that the $\hbox {SF}_4^{2+}$ has lost two fluorine atoms before dissociation to $\hbox {SF}_3^{+}$ + F^+^.

### Molecular dynamics and potential energy surface calculations

**Fig. 4:**
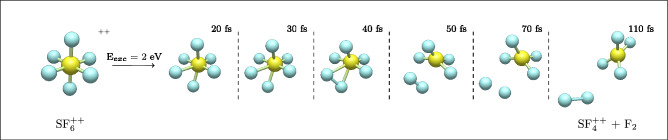
Snapshots from a representative molecular dynamics trajectory illustrating the structural evolution of $\hbox {SF}_6^{2+}$ over time. The images highlight key changes, ultimately leading to the formation of the most abundant channel found in the simulations: $\hbox {SF}_4^{2+}$ + F_2_.

To seek a deeper understanding of the putatively complex dissociative ionization mechanisms of SF_6_, we first performed molecular dynamics simulations, followed by a detailed exploration of the potential energy surfaces. Snapshots from a representative molecular dynamics trajectory when 2 eV of internal energy is deposited into initially octahedral doubly ionized SF_6_ in the ground state, shown in Fig. 4, illustrate the evolution of the system from the initial geometry to complete fragmentation at 110 fs. As this trajectory progresses, one of the S–F bond distances begins to increase, destabilizing the molecular structure. This elongation triggers the detachment of a neighboring fluorine atom, which subsequently recombines with the departing fluorine to form an F_2_ molecule. This ultrafast process is the predominant fragmentation channel suggested by the simulations, yielding $\hbox {SF}_4^{2+}$ and F_2_. Among the 160 simulations performed at different internal energies, this fragmentation pathway was found to be clearly the most probable, in agreement with the dominance of $\hbox {SF}_4^{2+}$ formation in the experimental results. The probabilities of other fragmentation channels, amounting to 20% of all the simulations, are presented in Table S1 of the Supplementary Materials.

**Fig. 5:**
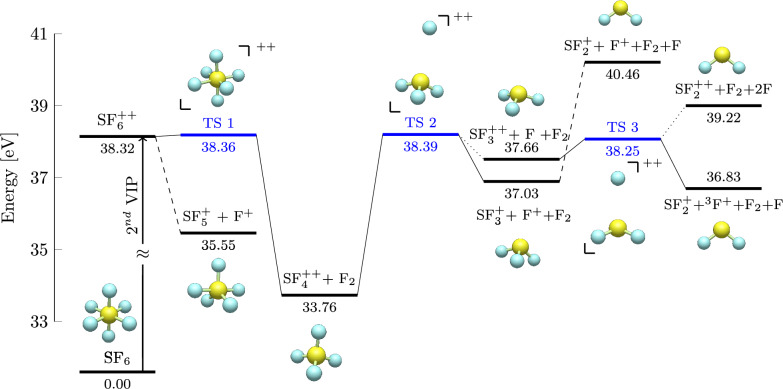
Potential energies for the dissociation of singlet $\hbox {SF}_6^{2+}$, calculated at the B3LYP/6-311+G(d) level of theory with zero-point energy (ZPE) corrections. The energies are given in eV relative to neutral SF_6_. The energy landscape of the dissociation processes is illustrated, highlighting transition states in blue and intermediates in black along the reaction pathways. Dashed lines indicate barrierless transitions, while dotted lines connect transition states to exit channels with higher energies due to different charge localization.

Following the molecular dynamics simulations, potential energy surface (PES) exploration, summarized in Fig. 5, was conducted. While MD simulations provide insight into the time-dependent evolution of dissociation, they do not explicitly characterize stationary points, such as structures of transition states and products or heights of energy barriers. Mapping the PES enabled the identification of critical points along the dissociation pathways, allowing for the determination of whether fragmentation proceeds via barrierless or activated processes.

In accordance with the lack of an intact doubly charged parent molecule in the mass spectra, theoretical calculations did not identify any stable form of $\hbox {SF}_6^{2+}$. Similarly, no stable structures of $\hbox {SF}_6^{+}$ or $\hbox {SF}_5^{2+}$ ions were found, reflecting their absence in the spectra. When looking for the first possible step of the fragmentation of a singlet $\hbox {SF}_6^{2+}$, a relaxed S-F bond scan revealed a transition state leading to the emission of a neutral F_2_ molecule from the dication. The transition state, labelled TS1 in Fig. 5, has reduced symmetry compared to the parent molecule because of a reduction in one of the F-S-F angles. The corresponding energy barrier was located only 0.04 eV above the calculated vertical ionization potential of SF_6_, suggesting immediate fragmentation to this channel upon ionization if the process proceeds on the ground electronic potential energy surface. Indeed, our experimental appearance energy for this fragment is just 37.4 eV. The discrepancy between this experimental appearance energy for $\hbox {SF}_4^{2+}$ and the calculated energy barrier of 38.36 eV may be attributed to the level of theory used in the calculations. Variational Kohn-Sham density-functional-theory (KS-DFT) has known limitations in precise barrier heights estimation^22^. While higher-level methods such as coupled-cluster single-double-(triple) (CCSD(T)) incorporate more accurate electron correlation effects, they are computationally significantly more expensive than DFT, making the latter a more practical choice for extensive PES exploration and for application of the statistical theory.

To investigate the mechanism of the main charge separation channel, a relaxed bond scan on $\hbox {SF}_4^{2+}$ was performed, revealing a transition state at an S-F bond length of 3.384 Å. Analysis of the Mulliken charges on structure TS2 in Fig. 5 indicates that this transition state leads to charge separation. Although the energy of the charge-retaining exit channel is lower than that of the transition state, it remains 0.63 eV higher than the charge separation channel. This energy difference further favours charge separation but does not preclude the formation of the $\hbox {SF}_3^{2+}$ fragment.

Further loss of F from the charge retaining fragment $\hbox {SF}_3^{2+}$ occurs via a small energy barrier, labelled TS3 at 38.25 eV. Interestingly, both the charge-retaining and charge-separation exit channels lie above this transition state. This feature suggests either (1) an additional, higher transition state may exist further along the reaction pathway (at a greater S–F bond distance), potentially exceeding the 40.46 eV exit channel energy of the charge-separation pathway, or (2) the transition state may lead to a local minimum where $\hbox {F}^{+}$ is in a triplet state, lying below the singlet state. The possibility of the first option is supported by the well-known tendency of DFT-B3LYP, used in this study, to yield inaccurate results at large interatomic distances, as well as by the experimentally observed high appearance energy of the $\hbox {SF}_2^{+}$ + $\hbox {F}^{+}$ pair at 44.0 eV. The second interpretation aligns with the discussion by Lange et al.^9^, who previously pointed out the possibility of producing triplet $\hbox {F}^{+}$ from $\hbox {SF}_4^{2+}$ via a spin-forbidden transition. In this system, strict spin conservation is not necessarily enforced, because spin–orbit coupling facilitates transitions between spin states.

The presence of a deep potential well and high energy barrier to further fragmentation of $\hbox {SF}_4^{2+}$ is consistent with metastable behaviour, as observed by Eland and Treeves-Brown^8^ and in all subsequent ion-ion coincidence studies of $\hbox {SF}_6^{2+}$. The internal energy remaining in some $\hbox {SF}_4^{2+}$ ions after their formation may be barely sufficient to overcome the fragmentation barrier, leading to slow dissociation, though tunnelling is unlikely. No energy barriers on the ground electronic potential energy surface have been found for the direct loss of $\hbox {F}^{+}$ from $\hbox {SF}_6^{2+}$, nor to further F loss from $\hbox {SF}_5^{+}$. The latter observation is consistent with the experimental finding that the $\hbox {SF}_4^{+}$ + F^+^ ion pair comes from F loss from $\hbox {SF}_5^{+}$.

**Table 2:** Fragmentation channels, calculated dissociation limits, enthalpies of reaction ($\Delta _r$H) and kinetic energy release (KER) of SF_6_ fragmentation upon double ionization. The theoretical appearance energies (AE) taken from Lange et al.^9^ and experimental thermodynamic thresholds based on data from NIST^23^ are also shown.

Ion(s)	Neutral(s)	Dissociation limit (eV)		AE (eV)^9^	$\Delta _r$H $\hbox {(eV)}^{\text {a}}$	$\Delta _r$H $\hbox {(eV)}^{\text {b}}$	KER $\hbox {(eV)}^{\text {a}}$
		B3LYP	CCSD(T)				
$\hbox {SF}_4^{2+}$	F_2_	33.76	33.02	33.30	33.82		4.6
	2F	35.08	34.46	34.99	35.17		
$\hbox {SF}_3^{2+}$	F_2_ + F	37.66	37.92	38.62	37.76		
	3F	38.98	39.35	40.31	39.12		
$\hbox {SF}_2^{2+}$	2F_2_	37.90	38.39	39.16	38.01		
	F_2_ + 2F	39.22	39.83		39.36		
$\hbox {SF}_3^{+}$ + F^+^	F_2_	37.03	36.19	34.23	37.13	33.86	1.36
	2F	38.35	37.63	35.91	38.48	35.50	
$\hbox {SF}_3^{+}$ + $\hbox {F}_2^+$	F	31.50	31.76	32.30	31.60	32.14	
$\hbox {SF}_2^{+}$ + F^+^	F_2_ + F	40.46	40.06	38.50	40.60	38.72	
	3F	41.77	41.50	40.18	41.96	40.37	
$\hbox {SF}_2^{+}$ + ($^3$P)F^+^	F_2_ + F	36.83	37.25				1.42
$\hbox {SF}_5^{+}$ + F^+^		35.55	33.76	31.33	35.60	31.08	2.77
$\hbox {SF}_4^{+}$ + F^+^	F	38.17	37.32	35.34	38.27	35.81	

$^{\text {a}}$Theoretical calculations in this work obtained at B3LYP/6-311+G(d) level of theory.

$^{\text {b}}$Calculated using formation enthalpies $\Delta _{f}H^{\circ }$(298 K) taken from NIST^23^ Database.

More understanding of dissociation mechanisms can be gained by analysis of kinetic energy releases (KERs) in relation to the details of the potential energy surface. The fraction of energy converted into translational motion of reaction products is directly influenced by the topology of the PES. Details of the current PES, including calculated and thermochemical dissociation limits and KER values, are presented in Table 2. Exit channels that are absent from Fig. 5, as they don’t follow the pathway of F_2_ loss from the parent ion are also included. Theoretical kinetic energy release values are determined by calculating the reverse activation barrier in cases where a transition state is present. For each charge-separation channel, the theoretical KER values are consistently lower than the experimental values reported in Table 1 and lower than the energies to be expected from Coulomb repulsion. This discrepancy may partially stem from the level of theory used in the calculations. However, in order to include Coulomb repulsion energy accurately, the surface would have to extend to an inter-charge distances of at least 100 Å.

**Fig. 6:**
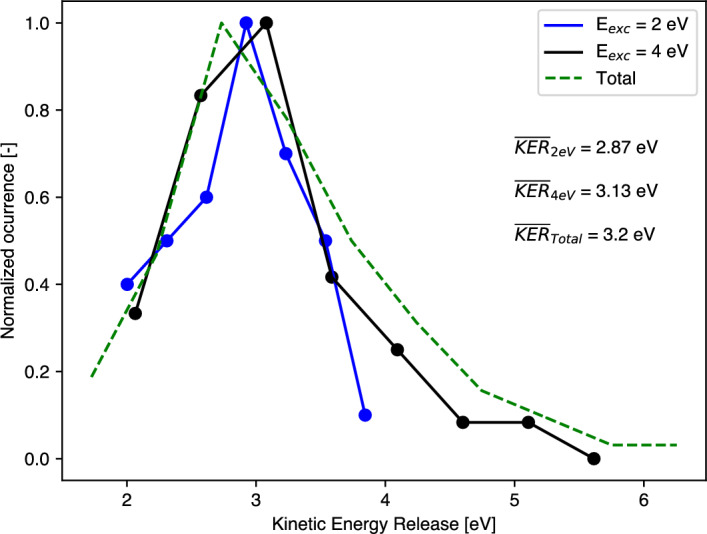
Theoretical kinetic energy release distribution in $\hbox {SF}_4^{2+}$ + F_2_. Solid lines refer to statistics for specific internal energy, while the dashed line shows the combined distribution from all 126 molecular dynamics trajectories with internal energies ranging from 2 to 8 eV in steps of 2 eV. Inset text provides average KER values.

If a reaction coordinate is entirely decoupled from other degrees of freedom, the reverse activation energy in that specific channel is fully converted into kinetic energy of the dissociating fragments. However, in many reactions of singly charged ions involving an activation barrier, experimentally observed kinetic energy release distributions are broader than theoretical predictions and are often shifted towards lower kinetic energies^24^. This can be because only a portion of the reverse activation energy is converted into translational motion, while the remainder is distributed into rotational and vibrational excitation of the products. In charge separation of doubly-charged ions, by contrast, most of the reverse activation energy (Coulomb repulsion energy) is released at separations where energy transfer to internal modes is very unlikely. In molecular dynamics simulations, we can go beyond PES analysis and extract kinetic energy releases directly from the translational motion of fragments. As most trajectories in these simulations lead to the production of $\hbox {SF}_4^{2+}$ with F_2_ we could investigate only this channel by this method. The resulting kinetic energy release distributions are presented in Fig. 6. The solid lines represent KER distributions for specific internal energy values, while the dashed line corresponds to the KER distribution obtained from all trajectories within this dissociation channel. The most probable KER value is approximately 3.0 eV, with average KER values of 2.87 eV, 3.13 eV, and 3.20 eV for internal energies of 2 eV, 4 eV, and the total KER, respectively. These average theoretical values are significantly higher than the experimental KER of less than 1 eV observed for $\hbox {SF}_4^{2+}$. Nevertheless, some further conclusions can be drawn from the shapes of distributions for different internal energies. At 2 eV, the distribution is relatively narrow, whereas at 4 eV, it exhibits a long tail characteristic of an exponential decay. It is noteworthy that increasing internal energy does not shift the most probable KER value but instead leads to more extensive energy partitioning into vibrational and rotational degrees of freedom. It may be a relevant observation that bimodal KER distributions have been reported before in decays of fluorine-containing singly-charged ions^25,26^.

**Fig. 7:**
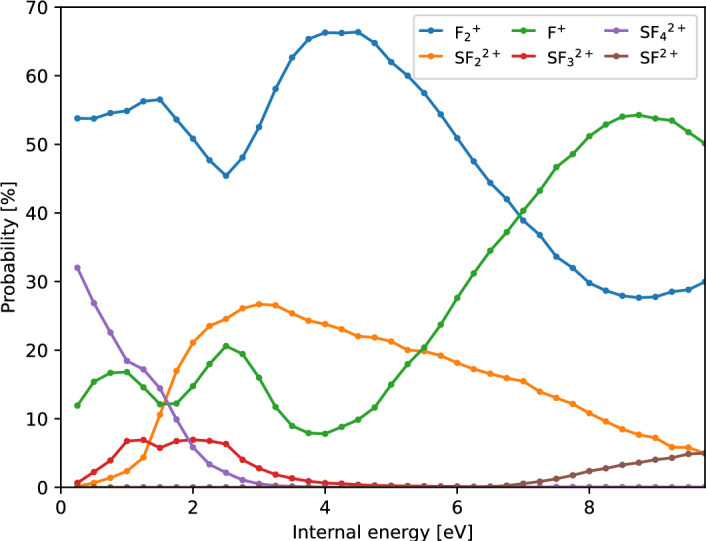
Breakdown diagram for the decay channels of $\hbox {SF}_6^{2+}$ obtained with M_3_C theory. Analogous to Fig. 3, F^+^ and $\hbox {F}_2^+$ are taken as proxies for the charge separating channels. The error bars, corresponding to the standard deviation in channel probabilities, are omitted for clarity, but are 9% of the quantities given, on average.

### Statistical calculations

Ab initio molecular dynamics simulations are a powerful tool for investigating the early stages of a fragmentation process. However, their high computational cost limits their applicability to short timescales, typically only a few hundred femtoseconds, small separation distances and small molecular systems. In contrast, statistical methods provide a computationally efficient alternative way to model fragmentation experiments. One such approach is the Microcanonical Metropolis Monte Carlo (M_3_C) method^13,14^, which allows for direct comparison of fragmentation breakdown curves with experimental results obtained by coincidence techniques. Recently, this methodology has been applied to reproduce the furan singly charged ion breakdown curves obtained by photoelectron-photoion coincidence (PEPICO) spectroscopy^27^. In view of its advantages, we here employ the M_3_C method to study the dissociation dynamics of doubly ionized $\hbox {SF}_{6}$, aiming for insights beyond the time limitations of MD simulations.

The M_3_C method models the fragmentation of an excited molecular system by distributing its mass, charge, energy, and momentum (both linear and angular) among all accessible fragmentation channels. This distribution follows the principles of the microcanonical ensemble, ensuring conservation of these physical quantities while determining the probability of each channel based on maximum entropy considerations. Using a Monte Carlo sampling approach, the system explores the phase space in a stochastic manner until it reaches a region of maximum entropy, where physical observables are obtained through statistical averaging. This methodology provides a computationally efficient framework for describing complex dissociation dynamics. However, it has inherent limitations, particularly in its inability to capture non-statistical dissociation processes and dynamical effects that depend on the potential energy surface topology.

Firstly, the M_3_C method assumes that the system obeys the ergodic hypothesis, meaning that over a sufficiently long period of time it explores all accessible microstates with equal probability. This implies that excess energy redistribution among internal degrees of freedom is statistical and efficient, leading to a fragmentation pattern dictated by the density of states and entropy maximization. Secondly, the M_3_C method neglects energy barriers based on the assumption that the excited molecular system possesses sufficient internal energy to overcome all potential barriers along the dissociation pathway. As a result, only the properties of the exit channels need to be considered and transition state dynamics are not explicitly modelled.

A critical requirement for the accuracy and reliability of this approach is the construction of a comprehensive database of possible fragments, ideally characterized using high-level quantum chemistry calculations. Each fragment is described by its molecular geometry, energy, vibrational frequencies, and symmetry properties, ensuring that the method correctly represents the accessible phase space. For highly symmetric molecules such as $\hbox {SF}_{6}$, the fragment database remains relatively small due to the limited number of distinct dissociation pathways. Additionally, isomerization processes, which can significantly increase the complexity of fragmentation databases for less symmetric systems, do not need to be considered in this case. As a result, the fragment database for singlet $\hbox {SF}_{6}^{2+}$ consists of 9 neutral, 9 singly ionized and 8 doubly ionized species, collected in Table S2 of the Supplementary Materials.

The primary result of an M_3_C calculation are the fragmentation channel probabilities and the relative abundances of species as a function of internal energy. Figure 7 presents the computed probabilities of charged species, with all channels producing $\hbox {F}^{+}$ or $\hbox {F}_2^{+}$ grouped together to facilitate direct comparison with the experimental data shown in Fig. 3. Fragmentation channel probabilities as a function of internal energy are shown in Fig. S2 of the Supplementary Materials. As expected, the statistical fragmentation model predicts a more complex dissociation pattern than that obtained from molecular dynamics simulations, highlighting the fundamental differences between the two approaches.

Fragmentation is observed from the lowest internal energy of 0.25 eV, with no detectable presence of the parent dication. This is consistent with experimental findings, where $\hbox {SF}_{4}^{2+}$ is the dominant product at low energies. A key discrepancy arises between the M_3_C model and the experimental results: in the statistical calculations, the probability of $\hbox {SF}_{4}^{2+}$ rapidly decreases with increasing internal energy and reaches zero around 3 eV. At the same time the abundance of all other dissociation channels rises immediately from near zero internal energy. In contrast, our experimental data show that $\hbox {SF}_{4}^{2+}$ remains the dominant species, maintaining a 100% yield up to 2.5 eV and all other channels gain intensity only at higher energies where $\hbox {SF}_{4}^{2+}$ diminishes. For the main charge-retaining channel, one possible explanation might be that in reality $\hbox {SF}_{4}^{2+}$ formation occurs by a non-statistical, ultrafast process, thereby outcompeting formation as assumed in the M_3_C model, which requires complete energy redistribution before fragmentation. Another notable discrepancy is the very high yield of $\hbox {F}_2^{+}$ predicted in the statistical calculations but not seen in experiment. This difference can be understood by considering the dissociation limits of competing fragmentation pathways. The fragmentation channel leading to $\hbox {SF}_{3}^{+}$/$\hbox {F}_2^{+}$/F has a threshold energy of 31.5 eV, whereas the alternative pathway forming $\hbox {SF}_{3}^{2+}$/F_2_/F requires 37.66 eV. Given these energetics, the M_3_C model predicts a higher entropic favorability for $\hbox {SF}_{3}^{+}$/$\hbox {F}_2^{+}$/F, as the approach does not incorporate information on the preceding potential energy surface. These results highlight the limitations of statistical methods. In reality, intramolecular vibrational redistribution and electronic state identity degradation may not always be efficient or sufficiently fast, leading to deviations from ergodicity. Non-ergodic behaviour of the cysteine-water clusters upon collision-induced activation has been shown before with the support of M_3_C results^28^ and we may have a further example here.

## Conclusions

The dissociations of $\hbox {SF}_{6}^{2+}$ ions have been investigated using a multi-particle ion-electron coincidence technique, combined with molecular dynamics simulations and calculations using the M_3_C fully statistical method. Nacent $\hbox {SF}_{6}^{2+}$ ions were formed by photoionization at 48.4 eV and their decay is found to be dominated at both the lowest and highest internal energies by an unusual abundance of double charge retaining reactions. Many characteristics of the dissociations are reproduced by molecular dynamics simulations starting on a ground-state potential energy surface, but an attempt to match the overall abundances of the final products by a purely statistical calculation falls short rather dramatically. We have to conclude that the statistical model is not appropriate here, because electronic energy degradation and/or internal vibration redistribution are not sufficiently complete or rapid in this doubly-charged molecule to precede dissociation. It seems possible that the molecular size at which ergodic behaviour becomes dominant is larger for doubly and multiply charged ions than for neutral and singly ionized molecules. A detailed theoretical investigation of the excited-state dynamics of $\hbox {SF}_6^{2+}$, particularly incorporating non-adiabatic effects, is essential for a more complete understanding of its fragmentation pathways. Advanced computational approaches, such as non-adiabatic molecular dynamics and multi-reference electronic structure methods, could provide critical insights into the role of electronic state crossings, avoided crossings, and charge redistribution in the dissociation process.

## Methods

### Experimental details

Multi-particle ion-electron coincidence experiments on SF_6_ were carried out at 256 Å (48.4 eV), i.e. about 11 eV above its known single-photon double ionization onset of 37.5 eV^1^. The multi-particle coincidence set-up used for the present work has been described before^29^. It allows simultaneous detection of, for instance, two electrons with known kinetic energies and two positive ions with known mass/charge. Higher numbers of ions and electrons can be detected, but since the triple ionisation energy of SF_6_ is far out of reach at the present photon energy all such events observed are treated as false coincidences.

The experimental setup consists of a magnetic bottle electron time of flight spectrometer, with a collection-detection efficiency of about 50% and a nominal resolving power of $E/ \Delta E = 20$, and a time of flight mass spectrometer, with a collection-detection efficiency of about 20% and a typical mass resolution for thermal ions of 1 mass unit at m/q = 100. A pulsed helium gas discharge lamp was used as source for ionizing radiation, providing photons at sufficiently high intensity at the emission lines of 21.2, 40.8 and 48.4 eV, which are wavelength selected individually using a toroidal grating monochromator.

The ionizing radiation intercepts the gas target molecules in the interaction region, where they are introduced into the system through a hollow gas needle in form of an effusive jet. A comparatively strong ring magnet with a conical pole piece guides the electrons into an 2.2 m long flight tube surrounded by a comparatively weak homogeneous solenoid field, where also a weak electric field ($\sim 1$ V) is applied across the interaction region to ensure that the low kinetic energy electrons are detected efficiently with usefully short fight times. After about 50 ns, when the electrons have left the interaction region, the ions are extracted in the opposite direction by a strong, pulsed electrical field and a static accelerating electrical field (in total $\sim 600$ V), optimized following the original two-field mass spectrometer concept of Wiley-McLaren^30^. The ions pass through the ring magnet into a 0.12 m long ion flight tube. The resulting flight times reflect the mass/charge of the ions, and from the time of flight peak widths we can deduce any kinetic energy in excess to the thermal kinetic energy. The KER from an ion dissociation can be estimated from the FWHM of the ion-ion coincidence peak and the electric field at the interaction point, a method which is likely to underestimate to some extent the actual KERs, thus providing primarily a lower limit^31^.

This apparatus allows us to measure complete mass spectra for single and double ionization separately. By selecting events which include only a single electron in the energy range of single ionisation, double ionisation is excluded. Contrariwise, by selecting events all of which include two electrons whose energy sum lies in the known double ionization range, we extract essentially pure double ionization data. Because of imperfect collection-detection efficiency and the presence of background electrons there is a small leakage of single ionization into the raw double ionization spectrum and a very small leakage of double ionization into single. Both of these can be allowed for in the data treatment by subtraction of a randomized electron pair spectrum. The double ionization spectra acquired in this way include both doubly-charged ions and the singly charged ion pairs produced by charge separation processes.

### Computational details

The theoretical approach employed in this study integrates three complementary techniques to analyze the fragmentation dynamics, energetic pathways, and entropic contributions of the investigated process. The first step involves conducting molecular dynamics (MD) simulations to determine dominant reaction mechanisms and probable final structures. *Ab initio* MD simulations were performed using the Atom-Centered Density Matrix Propagation (ADMP)^32–34^ method, coupled with the B3LYP^35,36^ functional and the 6-31G(d,p) basis set. The simulation time was limited to 300 fs with a 0.1 fs time step, ensuring accurate energy conservation and effective separation between electronic and nuclear motion. While fragmentation timescales are generally longer, a 300 fs window was sufficient to capture primary fragmentation trends of this doubly ionized system. Internal energies ranging from 2 to 8 eV in 2 eV increments were distributed randomly across nuclear degrees of freedom. Different initial velocity distributions facilitated independent trajectory sampling, leading to 40 distinct calculations per energy value, culminating in a total of 160 trajectories. To simulate experimental conditions, the calculations begin with the SF_6_ molecules in their ionized, electronic ground state, subjected to vertical excitation. The last step of each trajectory was analyzed to determine fragmentation channels, in which bond dissociation was identified when interatomic distances exceeded 2.5 Å.

To complement the MD results, the second step involved exploration of the $\hbox {SF}_6^{2+}$ potential energy surface (PES), focusing on the identification and characterization of key stationary points, including reactants, intermediates, transition states, and products. Geometry optimizations and harmonic vibrational frequency calculations were carried out using density functional theory (DFT) at the B3LYP/6-311+G(d) level. Dissociation limits have also been calculated at the coupled-cluster single-double-(triple) CCSD(T)/cc-pVTZ level of theory. The relative energies on the PES were adjusted to include the zero-point energy corrections. To verify transition state connectivity to their corresponding minima, intrinsic reaction coordinate (IRC) calculations were performed. This combined approach of DFT with ab initio MD, has proven highly effective in numerous prior investigations of molecular fragmentation of excited systems^27,37–39^.

The final step of our methodology relies on a statistical theory, where entropy maximization determines fragmentation branching ratios at a significantly lower computational cost than MD simulations by the assumption of the infinite integration time limit. The constrained Microcanonical Metropolis Monte Carlo (MMMC)^14,40^ approach, as implemented in the M_3_C code^13^, relies on the ergodic theorem to explore the available phase space until the region of maximum entropy is reached, allowing for a statistically robust fragmentation profile. The implementation of this methodology ensures conservation of the total angular momentum to zero by the sum of angular momenta from all fragments exactly compensating the orbital momentum^13^. To execute M_3_C simulations, a comprehensive database of potential fragments needs to be established. An automated isomer search was performed, initially optimizing geometries at the B3LYP/6-311+G(d) level before symmetrizing them at the same level of theory. This process identified 27 possible fragments, characterized by their geometry, electronic energy, frequencies and symmetry. Based on the convergence criteria of the average error (standard deviation) in channel probabilities being lower than 10%, parameters of the M_3_C simulations are selected. In the present work we have chosen the system radius as 7 Å, 10 000 steps in the Markov chain and 10 000 as the number of numerical experiments. The numerical experiments each begin with different, randomly selected values of vibrational energy, angular momentum, and molecular orientation. The initial structure is already ionized $\hbox {SF}_6^{2+}$.

All quantum chemistry simulations have been performed using the Gaussian16 software package^41^.

## Supplementary Information

Supplementary Information.

## Data Availability

The data sets generated during and/or analysed during the current study are available from the corresponding authors on reasonable request.

## Code availability

The computer codes customised during the current study are available from the corresponding authors on reasonable request.

## References

[1] Feifel, R., Eland, J. H. D., Storchi, L. & Tarantelli, F. Complete valence double photoionization of . *J. Chem. Phys.* **122**, 144309. 10.1063/1.1872837 (2005). 15847524 10.1063/1.1872837

[2] Christophorou, L. G. & Olthoff, J. K. Electron interactions with sf6. *J. Phys. Chem. Ref. Data* **29**, 267–330. 10.1063/1.1288407 (2000).

[3] Bull, J. N., Lee, J. W. L. & Vallance, C. Electron-impact-ionization dynamics of . *Phys. Rev. A* **96**, 042704. 10.1103/PhysRevA.96.042704 (2017).

[4] Kandhasamy, D. M., Albeck, Y., Jagtap, K. & Strasser, D. 3D coincidence imaging disentangles intense field double detachment of . *J. Phys. Chem. A* **119**(29), 8076–82 (2015). 26098224 10.1021/acs.jpca.5b04101

[5] Bapat, B., Sharma, V. & Kumar, S. V. K. Dissociative states of probed by fragment momentum spectroscopy. *Phys. Rev. A* **78**, 042503. 10.1103/PhysRevA.78.042503 (2008).

[6] Eland, J. The dynamics of three-body dissociations of dications studied by the triple coincidence technique PEPIPICO. *Mol. Phys.* **61**, 725–745. 10.1080/00268978700101421 (1987).

[7] Hsieh, S. & Eland, J. H. D. Charge separation reaction dynamics from PEPIPICO using a position-sensitive detector. *Rapid Commun. Mass Spectrom.* **9**, 1261–1265. 10.1002/rcm.1290091308 (1995).

[8] Eland, J. H. D. & Treves-Brown, B. J. Dissociative multiionization in molecules. *AIP Conf. Proc.* **258**, 100–117. 10.1063/1.42537 (1992).

[9] Lange, M., Pfaff, O., Müller, U. & Brenn, R. Projectile fragment–ion fragment–ion coincidences (PFIFICO) following fast ion impact on SF6. *Chem. Phys.* **230**, 117–141. 10.1016/S0301-0104(97)00378-9 (1998).

[10] Yencha, A. J., Lopes, M. C. A., Thompson, D. B. & King, G. C. Threshold photoelectron spectroscopy in the inner-valence ionization region and photo-double ionization of SF6. *J. Phys. B: At. Mol. Opt. Phys.* **33**, 945. 10.1088/0953-4075/33/5/310 (2000).

[11] Peterka, D. S., Ahmed, M., Ng, C.-Y. & Suits, A. G. Dissociative photoionization dynamics of by ion imaging with synchrotron undulator radiation. *Chem. Phys. Lett.* **312**, 108–114. 10.1016/S0009-2614(99)00918-5 (1999).

[12] Griffiths, W. & Harris, F. Single and double ionization energies of sulphur hexafluoride measured by double charge transfer spectroscopy. *Int. J. Mass Spectrom. Ion Process.* **85**, 259–264. 10.1016/0168-1176(88)83021-0 (1988).

[13] Aguirre, N. F., Díaz-Tendero, S., Hervieux, P.-A., Alcamí, M. & Martín, F. C: A computational approach to describe statistical fragmentation of excited molecules and clusters. *J. Chem. Theory Comput.* **13**, 992–1009. 10.1021/acs.jctc.6b00984 (2017). 28005371 10.1021/acs.jctc.6b00984

[14] Aguirre, N. F. et al. Fully versus constrained statistical fragmentation of carbon clusters and their heteronuclear derivatives. *J. Chem. Phys.* **150**, 144301. 10.1063/1.5083864 (2019). 30981259 10.1063/1.5083864

[15] Illenberger, E. & Momigny, J. Gaseous molecular ions. In *Topics in Physical Chemistry* **2**, 212 ff (Steinkopf Verlag Darmstadt, 1992).

[16] Simm, I., Danby, C. & Eland, J. The fragmentation of studied by photoelectron-photoion coincidence spectroscopy. *Int. J. Mass Spectrom. Ion Process.* **14**, 285 (1974).

[17] Holland, D. et al. An experimental and theoretical study of the valence shell photoelectron spectrum of sulphur hexafluoride. *Chem. Phys.* **192**, 333–353. 10.1016/0301-0104(94)00381-J (1995).

[18] Feil, S., Gluch, K., Scheier, P., Becker, K. & Märk, T. D. The anomalous shape of the cross section for the formation of SF3+ fragment ions produced by electron impact on SF6 revisited. *J. Chem. Phys.* **120**, 11465–11468. 10.1063/1.1753553 (2004). 15268180 10.1063/1.1753553

[19] Frasinski, L. J., Stankiewicz, M., Randall, K. J., Hatherly, P. A. & Codling, K. Dissociative photoionisation of molecules probed by triple coincidence; double time-of-flight techniques. *J. Phys. B: At. Mol. Phys.* **19**, L819. 10.1088/0022-3700/19/23/002 (1986).

[20] Joachims, H.-W., Rühl, E. & Baumgärtel, H. *BESSY Jahresbericht, BESSY GmbH*, Berlin (1986). An annual report which one can request from the synchrotron radiation research facility Bessy in Germany by sending an e-mail to “info@bessy.de”; for further information see also “www.bessy.de”.

[21] Kramida, A., Yu. Ralchenko, Reader, J. & and NIST ASD Team. NIST Atomic Spectra Database (ver. 5.12), [Online]. Available: https://physics.nist.gov/asd [2025, February 12]. National Institute of Standards and Technology, Gaithersburg, MD. (2024).

[22] Verma, P., Perera, A. & Bartlett, R. J. Increasing the applicability of DFT I: Non-variational correlation corrections from Hartree-Fock DFT for predicting transition states. *Chem. Phys. Lett.* **524**, 10–15. 10.1016/j.cplett.2011.12.017 (2012).

[23] NIST Chemistry WebBook, NIST Standard Reference Database number 69. [Online], 10.18434/T4D303 (2022).

[24] Laskin, J. & Lifshitz, C. Kinetic energy release distributions in mass spectrometry. *J. Mass Spectrom.* **36**, 459–478. 10.1002/jms.164 (2001). 11391803 10.1002/jms.164

[25] Gridelet, E. et al. Ground and excited state dissociation dynamics of ionized 1,1-difluoroethene. *J. Phys. Chem. A* **109**, 8225–8235. 10.1021/jp051542b (2005). 16834209 10.1021/jp051542b

[26] Harvey, J. *Threshold Photoelectron Spectra of Four Fluorinated Ethenes from the Ground Electronic State to Higher Electronic States* 111–142 (Springer International Publishing, Cham, 2014).

[27] Erdmann, E. et al. A general approach to study molecular fragmentation and energy redistribution after an ionizing event. *Phys. Chem. Chem. Phys.* **23**, 1859–1867. 10.1039/D0CP04890A (2021). 33439170 10.1039/d0cp04890a

[28] Tiefenthaler, L. et al. Non-ergodic fragmentation upon collision-induced activation of cysteine–water cluster cations. *Phys. Chem. Chem. Phys.* **25**, 5361–5371. 10.1039/D2CP04172C (2023). 36647750 10.1039/d2cp04172c PMC9930733

[29] Eland, J. H. D. & Feifel, R. Double ionisation of ICN and BrCN studied by a new photoelectron–photoion coincidence technique. *Chem. Phys.* **327**, 85–90. 10.1016/j.chemphys.2006.03.040 (2006).

[30] Wiley, W. C. & McLaren, I. H. Time-of-flight mass spectrometer with improved resolution. *Rev. Sci. Instrum.* **26**, 1150–1157. 10.1063/1.1715212 (1955).

[31] von Busch, F. Extraction of representative kinetic energy parameters from photofragmentation time-of-flight spectra. *J. Phys. B: At. Mol. Opt. Phys.* **34**, 431. 10.1088/0953-4075/34/3/318 (2001).

[32] Schlegel, H. B. et al. Ab initio molecular dynamics: Propagating the density matrix with Gaussian orbitals. *J. Chem. Phys.* **114**, 9758–9763. 10.1063/1.1372182 (2001).

[33] Iyengar, S. S. et al. Ab initio molecular dynamics: propagating the density matrix with Gaussian orbitals. II. Generalizations based on mass-weighting, idempotency, energy conservation and choice of initial conditions. *J. Chem. Phys.* **115**, 10291–10302. 10.1063/1.1416876 (2001).

[34] Schlegel, H. B. et al. Ab initio molecular dynamics: Propagating the density matrix with Gaussian orbitals. III. Comparison with Born-Oppenheimer dynamics. *J. Chem. Phys.* **117**, 8694–8704. 10.1063/1.1514582 (2002).

[35] Becke, A. D. Density-functional thermochemistry. III. The role of exact exchange. *J. Chem. Phys.* **98**, 5648–5652. 10.1063/1.464913 (1993).

[36] Lee, C., Yang, W. & Parr, R. G. Development of the Colle-Salvetti correlation-energy formula into a functional of the electron density. *Phys. Rev. B* **37**, 785–789. 10.1103/PhysRevB.37.785 (1988). 10.1103/physrevb.37.785 9944570

[37] Maclot, S. et al. Determination of energy-transfer distributions in ionizing ion-molecule collisions. *Phys. Rev. Lett.* **073201**, 1–6. 10.1103/PhysRevLett.117.073201 (2016). 10.1103/PhysRevLett.117.073201 27563959

[38] Piekarski, D. G. et al. Production of doubly-charged highly reactive species from the long-chain amino acid GABA initiated by Ar9+ ionization. *Phys. Chem. Chem. Phys.* **19**, 19609–19618. 10.1039/C7CP00903H (2017). 28393947 10.1039/c7cp00903h

[39] Erdmann, E., Łabuda, M., Aguirre, N. F., Díaz-Tendero, S. & Alcamí, M. Furan fragmentation in the gas phase: new insights from statistical and molecular dynamics calculations. *J. Phys. Chem. A* **122**, 4153–4166. 10.1021/acs.jpca.8b00881 (2018). 29543456 10.1021/acs.jpca.8b00881

[40] Díaz-Tendero, S., Hervieux, P.-A., Alcamí, M. & Martín, F. Statistical fragmentation of small neutral carbon clusters. *Phys. Rev. A* **71**, 033202. 10.1103/PhysRevA.71.033202 (2005).

[41] Frisch, M. J. *et al.* Gaussian16 Revision C.02 (Gaussian Inc. Wallingford CT, 2016).

