# Supplementary material for: Non-ergodic dissociative valence double ionization of SF6
Source: Sci Rep. 2025 Jul 1;15:20751. doi: 10.1038/s41598-025-06972-0 (PMC12216322; doi:10.1038/s41598-025-06972-0)
Supplement: Supplementary file 1 — Supplementary Information. [file 41598_2025_6972_MOESM1_ESM.pdf]

Supplementary Materials: Non-ergodic dissociative valence double ionization of SF_6

Emelie Olsson[1], Veronica Daver Ideböhn[1], Måns Wallner[1], Richard J. Squibb[1], John H.D. Eland[2], Ewa Erdmann[3*], and Raimund Feifel[1,]**

[1]University of Gothenburg, Department of Physics, Origovägen 6B, 412 58 Gothenburg, Sweden
[2]Oxford University, Department of Chemistry, Physical and Theoretical Chemistry Laboratory, South Parks Road, Oxford OX1 3QZ, United Kingdom
[3]Faculty of Applied Physics and Mathematics, Gdańsk University of Technology, Narutowicza 11/12, 80-233 Gdańsk, Poland
[*]corresponding author: ewa.erdmann@pg.edu.pl
[**]corresponding author: raimund.feifel@physics.gu.se

Table S1. Molecular dynamics results: fragmentation channel probabilities in % at the final step of the simulation for different energy values deposited into SF_6^{2+}.

Channel	Internal Energy [eV]			
	2	4	6	8
$SF_4^{2+} + F_2$	97.5	92.5	82.5	42.5
$SF_5^{+} + F^{+}$	2.5	7.5	7.5	12.5
$SF_3^{+} + F_3^{+}$			2.5	2.5
$SF_3^{+} + F_2^{+} + F$			7.5	37.5
$SF_2^{+} + F_2^{+} + F_2$				5

Table S2. Fragments database for M_3C simulations. For each fragment charge, multiplicity, symmetry and electronic energy are given.

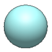<br>1<br>F.q0.m2-1<br>2-P (R3)<br>−99.76058 Ha	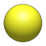<br>2<br>S.q0.m1-1<br>1-D (R3)<br>−398.07155 Ha	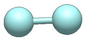<br>3<br>F2.q0.m1-9<br>1-SGG (D*H)<br>−199.57174 Ha	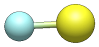<br>4<br>SF.q0.m2-9<br>2-PI (C*V)<br>−498.01506 Ha	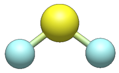<br>5<br>SF2.q0.m1-9<br>1-A1 (C2V)<br>−597.86390 Ha
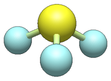<br>6<br>SF3.q0.m2-9<br>2-A1 (C3V)<br>−697.65409 Ha	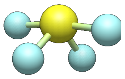<br>7<br>SF4.q0.m1-9<br>1-A1 (C2V)<br>−797.62084 Ha	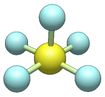<br>8<br>SF5.q0.m2-3<br>2-A1 (C4V)<br>−897.42590 Ha	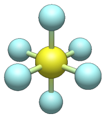<br>9<br>SF6.q0.m1-5<br>1-A1G (OH)<br>−997.31156 Ha	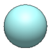<br>10<br>F.q1.m1-1<br>1-D (R3)<br>−98.97424 Ha
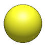<br>11<br>S.q1.m2-1<br>2-D (R3)<br>−397.66389 Ha	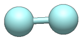<br>12<br>F2.q1.m2-9<br>2-PIG (D*H)<br>−198.98906 Ha	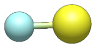<br>13<br>SF.q1.m1-9<br>1-PI (C*V)<br>−497.57473 Ha	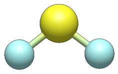<br>14<br>SF2.q1.m2-9<br>2-B1 (C2V)<br>−597.50702 Ha	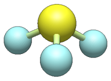<br>15<br>SF3.q1.m1-9<br>1-A1 (C3V)<br>−697.39654 Ha
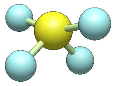<br>16<br>SF4.q1.m2-6<br>2-A1 (C2V)<br>−797.16574 Ha	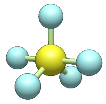<br>17<br>SF5.q1.m1-5<br>1-A1' (D3H)<br>−897.02807 Ha	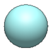<br>18<br>F.q2.m2-1<br>2-D (R3)<br>−97.61770 Ha	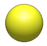<br>19<br>S.q2.m1-1<br>1-S (R3)<br>−396.81737 Ha	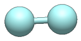<br>20<br>F2.q2.m1-9<br>1-PIG (D*H)<br>−197.85292 Ha
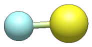<br>21<br>SF.q2.m2-9<br>2-PI (C*V)<br>−496.84186 Ha	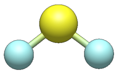<br>22<br>SF2.q2.m1-7<br>1-A1 (C2V)<br>−596.76610 Ha	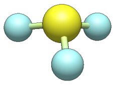<br>23<br>SF3.q2.m2-2<br>2-A1 (C3V)<br>−696.58694 Ha	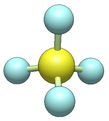<br>24<br>SF4.q2.m1-5<br>1-A1 (TD)<br>−796.49607 Ha	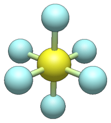<br>25<br>SF6.q2.m1-1<br>1-AG (CI)<br>−995.89768 Ha

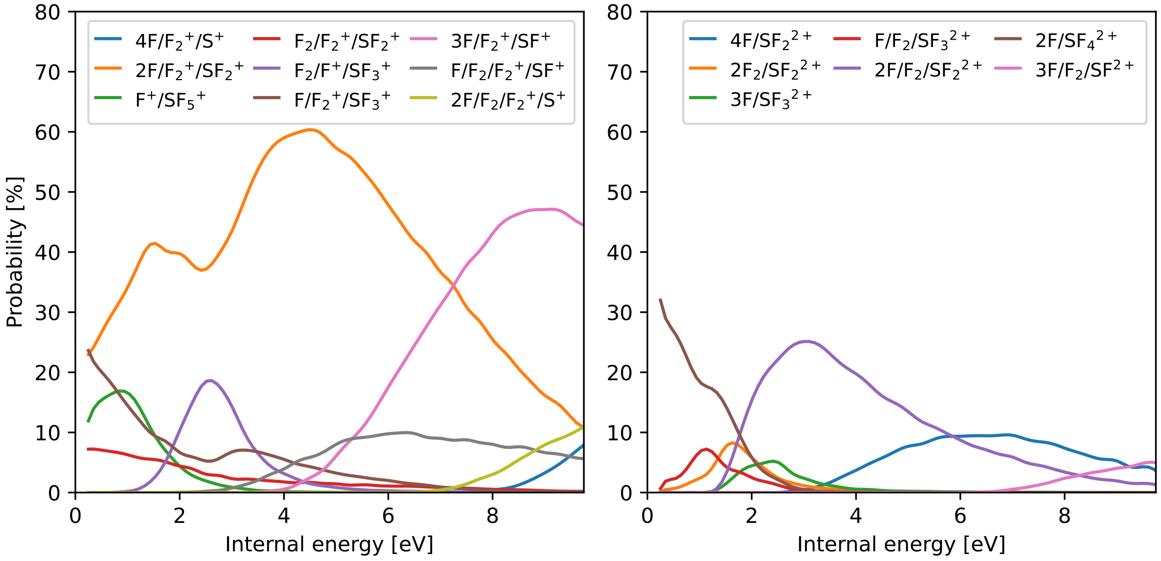

Figure S1. Decay channels of SF_6^{2+} obtained with M_3C theory. Left panel collects channels of charge separation, while right panel shows charge retaining channels. The error bars, corresponding to the standard deviation in channel probabilities, are omitted for clarity, but are 9 % of the quantities given, on average.
